# Influence of Failure to Rescue on Mortality After Transcatheter Aortic Valve Replacement

**DOI:** 10.1016/j.atssr.2025.03.011

**Published:** 2025-04-03

**Authors:** Muath Bishawi, Christopher Jensen, Andrew Vekstein, Andrzej S. Kosinski, Fred L. Grover, J. Kevin Harrison, Vinod H. Thourani, Ajay J. Kirtane, Joseph E. Bavaria, Sreekanth Vemulapalli, G. Chad Hughes

**Affiliations:** 1 Division of Cardiovascular and Thoracic Surgery, Duke University Medical Center, Durham, North Carolina; 2 Duke Clinical Research Institute, Duke University School of Medicine, Durham, North Carolina; 3 Department of Biostatistics and Bioinformatics, Duke University Medical Center, Durham, North Carolina; 4 University of Colorado School of Medicine, Denver, Colorado; 5 Division of Cardiology, Duke University Medical Center, Durham, North Carolina; 6 Piedmont Heart Institute, Atlanta, Georgia; 7 Division of Cardiology and the Cardiovascular Research Foundation, Columbia University Irving Medical Center/NewYork-Presbyterian Hospital, New York, New York; 8 Division of Cardiovascular Surgery, University of Pennsylvania, Philadelphia, Pennsylvania

## Abstract

**Background:**

Mortality after transcatheter aortic valve replacement (TAVR) varies among centers. “Failure to rescue” (FTR) patients from post-TAVR complications may represent an unexplored opportunity for TAVR process improvement.

**Methods:**

The Society of Thoracic Surgeons/American College of Cardiology Transcatheter Valve Therapy registry was queried for patients undergoing transfemoral TAVR between 2011 and 2016. Hospital FTR rate was derived from the ratio of observed-to-expected procedural mortality. Multivariable logistic regression models assessed the association between FTR and hospital mortality. Adjusted FTR rates were compared across tertiles of hospital mortality.

**Results:**

The analysis included 61,804 patients (429 sites). Post-TAVR mortality at low-, middle-, and high-mortality hospitals was 1.8%, 3.3%, and 5.6% (*P* < .01), respectively. Risk-adjusted complication rates differed only slightly between tertiles (22.2% vs 24.5% vs 27.0%, *P* < .001). However, adjusted FTR rates were significantly worse in high- and medium-mortality hospitals than in low-mortality centers (14.6% vs 9.5% vs 5.4%, *P* < .001). This was true for all investigated complications, including conversion to open surgery (high-mortality: odds ratio [OR], 9.04 [95% CI, 4.12-19.83], *P* < .001; medium-mortality: OR 2.99 [95% CI, 1.48-6.07], *P* < .003), stroke (high-mortality: OR, 3.15 [95% CI, 1.97-5.04], *P* < .001; medium-mortality: OR, 1.67 [95% CI, 1.05-2.67], *P* < .032), and cardiac arrest (high-mortality: OR, 3.54 [95% CI, 2.57-4.87], *P* < .001; medium-mortality: OR, 1.67 [95% CI, 1.24-2.24], *P* < .001).

**Conclusions:**

National TAVR mortality rates vary significantly across centers, despite comparable rates of postoperative complications. Patients at medium- and high-mortality centers face a disproportionately higher risk of death due to FTR. These findings highlight the need for a closer evaluation of post-TAVR care processes to address this disparity.

## Visual Abstract

**Figure:**
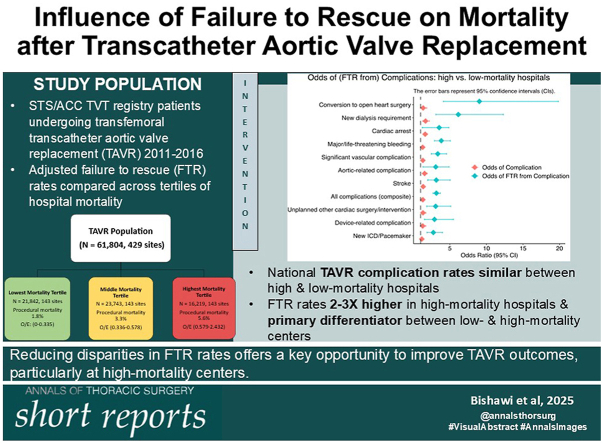

**In Short**

▪ National transcatheter aortic valve replacement (TAVR) mortality rates vary widely across centers, despite similar postoperative complication rates.
▪ Failure to rescue (FTR) is a quality metric defined as the inability to prevent death from complications. Despite similar complication rates, hospitals with higher mortality after TAVR exhibit significantly worse FTR rates, indicating a critical gap in postprocedural management.
▪ Reducing disparities in FTR rates offers a key opportunity to improve TAVR outcomes, particularly at high-mortality centers.

The number of centers performing transcatheter aortic valve replacement (TAVR) has steadily increased, accompanied by significant variation in hospital mortality rates, partly driven by differences in hospital volume.^1^ Notably, the inverse relationship between volume and mortality persists, even after adjusting for patient baseline risk factors, suggesting the influence of additional mechanisms beyond differences in case-mix.

Failure to rescue (FTR) is a quality metric defined as failure to prevent death from complications developing after hospital admission or an inpatient procedure.^2^ Prior data have shown that postoperative complication rates are similar across hospitals, yet mortality rates vary widely. FTR rates are strongly correlated with risk-adjusted postoperative mortality, suggesting that the inability to rescue a patient from a complication, rather than the complication itself, serves as an independent driver of postoperative mortality.^3^

Data on differences in post-TAVR complication rates and FTR are limited, as is their potential role in explaining variations in post-TAVR mortality across centers. Although some complications may not be preventable, the ability to rapidly diagnose and rescue a patient from a complication relates to a health care system’s quality of care,^4^ and differences in FTR rates may highlight potentially important areas for TAVR outcome improvement. To address this gap in the literature, the current study examined national FTR rates after transfemoral TAVR, with a focus on the relationship between hospital-specific mortality rates and FTR.

## Patients and Methods

Patients receiving a commercially approved TAVR device are reported to The Society of Thoracic Surgeons (STS)–American College of Cardiology (ACC) Transcatheter Valve Therapy (TVT) Registry according to Centers for Medicare and Medicaid Services guidelines. The National Cardiovascular Data Registry and Duke Clinical Research Institute house the data and perform quality checks, including random data audits of participating sites.^1^ The Duke University Medical Center Institutional Review Board approved this study and granted waiver of informed consent (Pro #00106459).

The study population included all STS-ACC TVT Registry patients undergoing transfemoral TAVR for primary aortic stenosis or mixed stenosis/insufficiency between 2011 and 2016. Patients with prior surgical aortic valve replacement, valve-in-valve TAVR, or missing in-hospital complication/procedural mortality data were excluded, as were sites with <10 patients.

Procedural mortality was defined as all in-hospital or postdischarge deaths through the follow-up assessment date within 75 days of TAVR. Consistent with other TVT registry studies, a 75-day window was chosen to ensure adequate collection of 30-day follow-up data.^5^ FTR from a complication was defined as death of a patient with a given complication, whereas the FTR rate for a given complication was calculated as number of FTR events divided by number of patients with the complication.

The in-hospital complications evaluated are detailed in the Supplemental Material and defined according to Valve Academic Research Consortium guidelines.^6^ Composite outcome variables were generated for aortic-related and device-related complications and are detailed in the Supplemental Material. All outcomes were defined in accordance with STS/ACC TVT definitions.^5^

The unadjusted mortality rate for each hospital was calculated as the number of procedural deaths divided by the total number of patients treated. The expected number of deaths at each hospital was estimated using the STS predicted risk of mortality score, which was then used to calculate the observed-to-expected (O/E) mortality ratio. Hospitals were subsequently divided into 3 groups according to tertiles of the O/E ratio.

Baseline characteristics and complication rates were compared across hospital mortality tertiles. Continuous and categorical variables are summarized as medians with interquartile ranges and counts with percentages, respectively. Observed complication and FTR rates were calculated within each hospital mortality tertile and compared across tertiles using the Wilcoxon rank sum test.

Unadjusted and risk-adjusted associations between hospital mortality tertiles and each individual complication were assessed using logistic regression models, with details in the Supplemental Material. Covariates in risk-adjusted models are likewise detailed in the Supplemental Material.

Unadjusted and risk-adjusted associations between hospital mortality tertiles and FTR rates for each investigated complication were assessed by logistic regression as detailed in the Supplemental Material. All statistical analyses were performed using R 4.1.1 software (R Foundation for Statistical Computing), with designated significance threshold of ≤.05.

## Results

**Figure 1:**
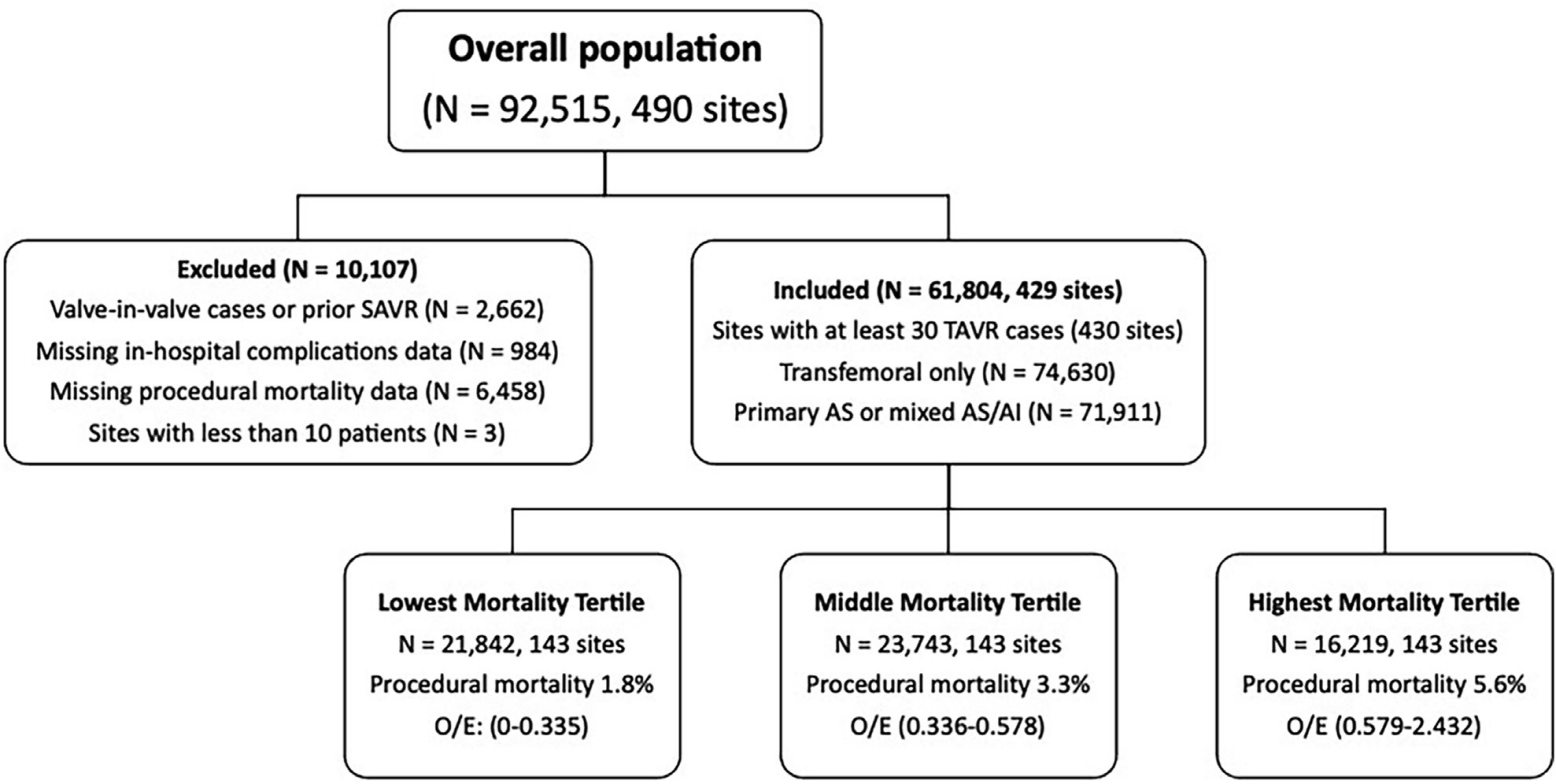
Study flow diagram. Transcatheter aortic valve replacement (TAVR) centers were grouped into tertiles of procedural mortality. (AI, aortic insufficiency; AS, aortic stenosis; O/E, observed/expected; SAVR, surgical aortic valve replacement.)

Inclusion criteria were met by 61,804 patients across 429 sites (Figure 1). Each mortality tertile consisted of 143 centers. There were 21,842 patients in the lowest mortality tertile, with an O/E range of 0 to 0.335; 23,743 in the middle tertile, with an O/E range of 0.336 to 0.578; and 16,219 in the highest tertile, with an O/E range of 0.579 to 2.432. Patient baseline characteristics across institutional mortality tertiles were similar (Supplemental Table 1). Procedural mortality for the lowest, middle, and highest mortality tertiles was 1.8%, 3.3%, and 5.6%, respectively (*P <* .001) (Supplemental Table 2).

**Figure 2:**
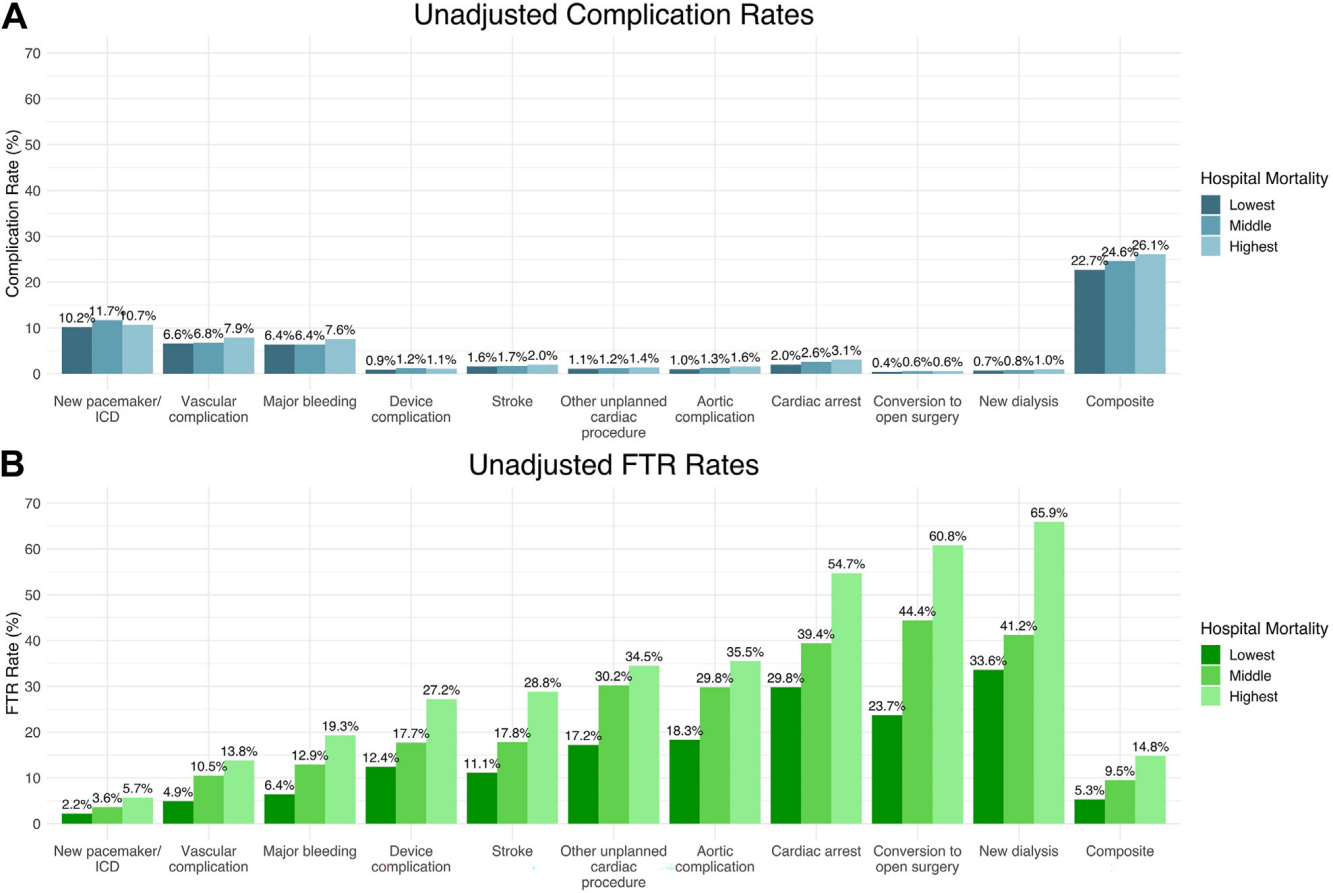
Unadjusted complication and failure to rescue (FTR) rates across hospital mortality tertiles. High-mortality transcatheter aortic valve replacement (TAVR) centers (A) do not have consistently higher complication rates but (B) do have significantly higher unadjusted rates of FTR once complications develop. (ICD, implantable cardioverter defibrillator.)

The differences in observed complication rates between hospital mortality tertiles were small and not clinically meaningful (Supplemental Table 3; Figure 2A). However, unadjusted rates of FTR from each complication differed greatly between hospital tertiles (Supplemental Table 4; Figure 2B). For example, the rate of conversion to open surgery was 0.4%, 0.6%, and 0.6% for low-, middle-, and high-mortality hospitals, respectively (*P* = .012). However, the corresponding FTR from this complication by lowest to highest mortality tertile was 23.7%, 44.4%, and 60.8% (*P <* .001). Furthermore, higher FTR rates correlated with increasing hospital mortality for all investigated adverse outcomes (Figure 2B): aortic-related complications (18.3% vs 29.8% vs 35.5%, *P <* .001), device-related complications (12.4% vs 17.7% vs 27.2%, *P <* .001), new dialysis (33.6% vs 41.2% vs 65.9%, *P <* .001), unplanned additional interventions (17.2% vs 30.2% vs 34.5%, *P <* .001), major bleeding (6.4% vs 12.9% vs 19.3%, *P <* .001), and the composite of all outcomes (5.3% vs 9.5% vs 14.8%, *P <* .001). For all examined complications, FTR rates in the highest-mortality hospitals were 2- to 3-times higher than in the lowest-mortality hospitals. Especially lethal complications associated with FTR rates >50% at high-mortality institutions included conversion to open surgery (60.8%), cardiac arrest (54.7%), and new dialysis requirement (65.9%) (Figure 2B).

**Figure 3:**
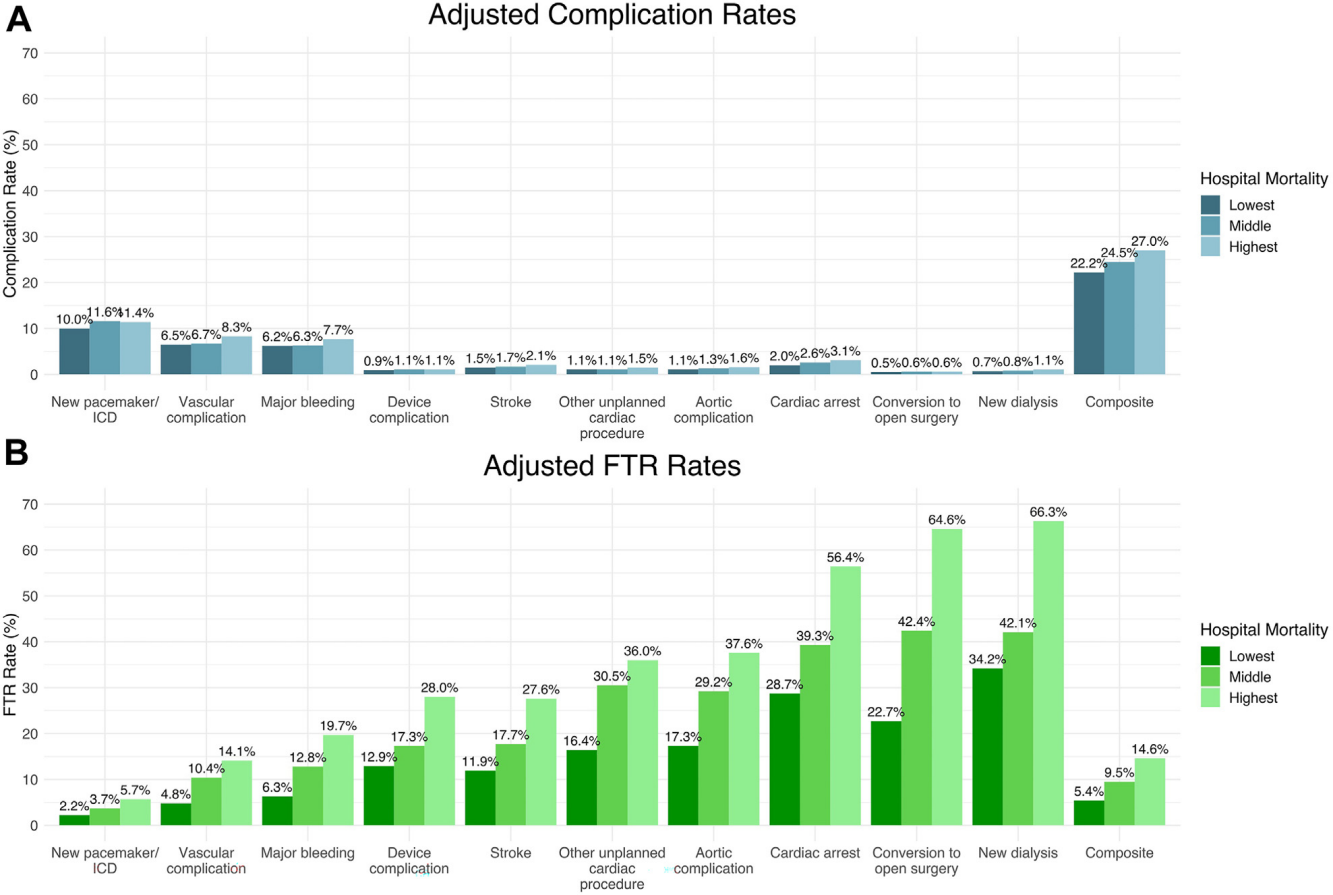
Adjusted complication and failure to rescue (FTR) rates across hospital mortality tertiles. (A) High-mortality transcatheter aortic valve replacement (TAVR) centers have slightly higher adjusted complication rates. (B) However, this trend is dwarfed by differences in adjusted FTR rates across mortality tertiles. (ICD, implantable cardioverter defibrillator.)

**Figure 4:**
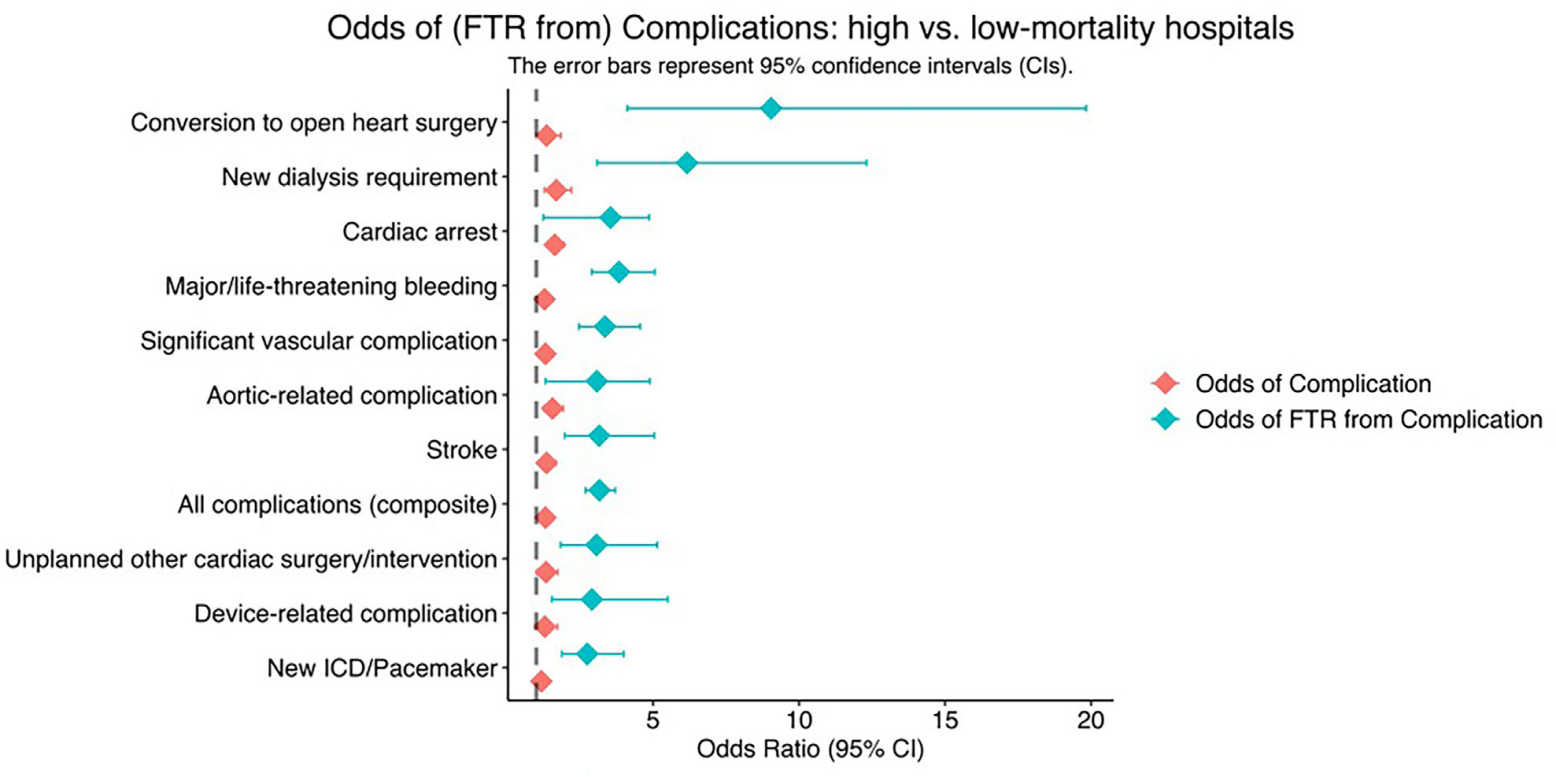
Patients treated at high-mortality transcatheter aortic valve replacement (TAVR) centers are significantly more likely to die of complications when they occur. (FTR, failure to rescue; ICD, implantable cardioverter defibrillator.)

These findings persisted after adjusting for underlying patient and hospital characteristics. Differences in adjusted complication rates between hospital mortality tertiles remained small and not clinically meaningful (Supplemental Table 5; Figure 3A). By contrast, FTR rates for most complications worsened after statistical adjustment (Supplemental Table 6; Figure 3B). This was especially apparent when FTR rates between high and low-mortality centers were compared; for example, the unadjusted FTR after conversion to open surgery was 4.99 but increased to 9.04 after adjustment (Supplemental Table 6). Additionally, patients at high-mortality centers were more likely to die after major vascular complications (odds ratio [OR], 3.35; *P <* .001), post-TAVR stroke (OR, 3.15; *P <* .001), requiring new dialysis (OR, 6.16; *P <* .001), undergoing unplanned cardiac intervention (OR, 3.06; *P <* .001), experiencing major bleeding (OR, 3.83; *P <* .001), as well as after a composite of all complications (OR, 3.16; *P <* .001) (Figure 4). Furthermore, differences in adjusted complication rates (Supplemental Table 5) and FTR (Supplemental Table 6) were minimally impacted by other institutional factors such as high vs low institutional TAVR volume, teaching vs nonteaching hospital, and hospital bed volume.

## Comment

This study analyzed data from the STS/ACC TVT registry to assess risk-adjusted differences in complication and FTR rates across United States centers performing TAVR. The key finding was that although postprocedural complication rates varied only slightly, there was substantial variability in the ability of centers to rescue patients (ie, “failure to rescue”) from these complications. This variability was strongly correlated with mortality rates and was the primary differentiator between the lowest- and highest-mortality centers. Notably, no association was found between FTR rates and factors traditionally considered important for TAVR outcomes, such as institutional TAVR volume, teaching hospital status, or hospital bed capacity.^1^ These findings suggest that other, currently unidentified factors influence a center’s ability to successfully manage complications once they occur.

A number of post-TAVR complications demonstrated significant variability in FTR rates across hospitals, with the most pronounced disparity observed in conversion to open surgery—a rare complication occurring in only 0.4% to 0.6% of cases across mortality tertiles. Patients at high-mortality centers were >9-times more likely to die of this complication compared with those at low-mortality centers. Similar trends were noted for many other complications, including cardiac arrest and new dialysis. These differences are likely multifactorial, potentially reflecting variations in the speed of diagnosis and deployment of secondary interventions.

The absence of a relationship between institutional TAVR volume and FTR rates is particularly unexpected, because it seems reasonable to assume that high-volume centers, benefitting from greater accumulated experience, would be better equipped to quickly identify and manage complications. However, adjusted analyses revealed no significant association between volume and FTR rates. This finding contrasts with other surgical procedures, where higher institutional volume is typically associated with lower FTR rates, suggesting that TAVR may be unique in this regard.^7^

The lethality of conversion to open surgery highlights potential differences in surgical expertise between centers. Some high-volume TAVR programs may lack equivalent experience or quality in cardiac surgery, possibly impacting outcomes. Additionally, variations in the functionality of “heart teams”—with better outcomes potentially linked to greater surgeon engagement in TAVR programs—may also contribute. These findings underscore the need for further research, and future studies could use the recently developed STS/ACC TVT Registry Composite Metric or other surgical quality measures to identify programmatic factors associated with optimized FTR rates.^8^

This study has several limitations. As with all large multicenter databases, the data are highly reliant on reporting quality at the center level, although extensive efforts have been made to standardize reporting within the STS/ACC TVT Registry.^9^

Second, the STS predicted risk of mortality score is a validated tool but may not fully capture patient-specific risks unique to TAVR. However, prior studies have demonstrated its correlation with post-TAVR survival.^10^

Finally, although this analysis highlights significant differences in outcomes between centers, it does not identify the underlying causes. Variability in TAVR care pathways, policies, and hospital-specific procedures likely contribute to differences in FTR rates. Although care pathways are partially standardized through Centers for Medicare and Medicaid Services guidelines and existing standard of care, further research is needed to identify clinical protocols or other unknown factors that significantly influence FTR rates.

In conclusion, FTR is an important health care quality metric that directly influences procedure-related mortality at the hospital level. This analysis of 61,804 transfemoral TAVR patients across 429 sites in the STS/ACC TVT Registry observed significant differences in FTR rates between hospitals in the highest and lowest mortality tertiles. These findings underscore the need for further investigation into the institutional factors that contribute to FTR variability and offer a significant opportunity to improve outcomes and enhance quality of care at lower-performing centers.
