# Supplementary material for: Influence of Failure to Rescue on Mortality After Transcatheter Aortic Valve Replacement
Source: Ann Thorac Surg Short Rep. 2025 Apr 3;3(3):617–23. doi: 10.1016/j.atssr.2025.03.011 (PMC12559580; doi:10.1016/j.atssr.2025.03.011)
Supplement: Supplementary Material [file mmc1.docx]

**Supplementary Material**

**Supplementary Methods**

*Outcomes measured*

In-hospital complications evaluated included: new permanent pacemaker or implantable cardioverter-defibrillator (ICD); conversion to open surgery; unplanned other cardiac surgery or intervention; stroke; cardiac arrest; aortic valve reintervention; new dialysis; and major/life-threatening bleeding or vascular complication.

A composite outcome variable was generated for aortic-related complications, defined as any of the following: annular rupture, aortic dissection, or perforation with or without tamponade. Similarly, a composite for device-related complications included any of the following: device migration, including migration into the left ventricle or aortic embolization, device recapture or retrieval, device thrombosis, or other device-related events.

*Statistical Methods*

Unadjusted and risk-adjusted associations between hospital mortality tertiles and each individual complication were assessed using logistic regression models. A random intercept for hospital was included to account for potential correlations among patients clustered within each hospital. Odds ratios with 95% confidence intervals (CIs) for each mortality tertile were calculated. Risk-adjusted complication rates were estimated for each tertile by averaging the predicted probability of the complication over the entire study cohort under a counterfactual assumption that all the patients belong to that tertile. Missing data on covariates was handled with single imputation, *i.e.*, missing values are imputed to median for continuous variables and to mode for categorical variables (with missing values for binary risk factors imputed to “No”).

Covariates in risk-adjusted models included: hospital total TAVR volume; teaching hospital status; hospital bed size (0-500 vs. 501-1000 vs. >1000); age; gender; gender-specific body surface area; left ventricular ejection fraction; hemoglobin; platelet count; procedure date; glomerular filtration rate; current dialysis; non-white or Hispanic; left main stenosis ≥50%; proximal left anterior descending stenosis ≥70%; prior myocardial infarction; endocarditis; prior stroke or transient ischemic attack; carotid stenosis; peripheral arterial disease; current/recent smoker; diabetes; NYHA class IV heart failure; atrial fibrillation/flutter; conduction defect; severe chronic lung disease; home oxygen; hostile chest; porcelain aorta; pacemaker; previous ICD; prior percutaneous coronary intervention; prior coronary bypass grafting; prior cardiac operation (2+ vs. 1 vs. 0); prior aortic valve procedure; prior non-aortic valve procedure; aortic valve disease etiology (degenerative vs. other); valve morphology (tricuspid vs. other); moderate/severe aortic insufficiency; moderate/severe mitral insufficiency; moderate/severe tricuspid insufficiency; acuity of TAVR (elective vs. urgent vs. shock or inotropes or assist device vs. emergency or salvage or cardiac arrest).

Unadjusted and risk-adjusted associations between hospital mortality tertiles and FTR rates for each investigated complication were assessed by logistic regression. A random intercept for hospital was included to account for potential correlations among patients clustered within each hospital. Odds ratios with 95% CIs for each mortality tertile compared to the lowest tertile were presented. Risk-adjusted FTR rates were calculated for each tertile by averaging the predicted probability of complication-specific FTR among all patients with that complication under a counterfactual assumption that all of them belong to that tertile. Covariates above were included in the risk-adjusted models. Missing covariate data was likewise handled by single imputation. As mortality tertile was considered ordinal, continuous variables were assessed using Spearman rank correlation coefficient, and categorical variables were compared using Wilcoxon rank sum test or Kruskal-Wallis test.

**Supplementary Table 1:** Baseline characteristics stratified by hospital mortality tertile.

|  | **Overall  N=61,804** | **Lowest Tertile  N=21,842** | **Middle Tertile  N=23,743** | **Highest Tertile  N=16,219** | ***p*-value** |
| --- | --- | --- | --- | --- | --- |
| **Age, years** | 83.0(77.0-87.0) | 83.0(77.0-87.0) | 83.0(77.0-88.0) | 83.0(77.0-88.0) | <.001 |
| **Male** | 32,894(53.2%) | 11,555(52.9%) | 12,512(52.7%) | 8,827(54.4%) | 0.008 |
| **White** | 58,041(94.7%) | 20,620(95.1%) | 22,304(94.7%) | 15,117(94.2%) | <.001 |
| **STS PROM, %** | 6.0(3.9-9.2) | 6.1(4.0-9.5) | 6.0(3.9-9.2) | 5.7(3.8-8.7) | <.001 |
| **Body surface area, m^2^** | 1.9(1.7-2.0) | 1.9(1.7-2.0) | 1.9(1.7-2.0) | 1.9(1.7-2.0) | 0.850 |
| **Hemoglobin, g/dL** | 11.9(10.6-13.1) | 11.9(10.7-13.1) | 11.9(10.6-13.1) | 11.9(10.6-13.1) | 0.013 |
| **Platelet count, K/µL** | 193.0(154.0-239.0) | 193.0(155.0-239.0) | 193.0(154.0-238.0) | 191.0(154.0-238.0) | 0.022 |
| **GFR (MDRD equation)** | 60.9(45.0-76.9) | 60.9(44.9-76.8) | 60.9(45.1-76.8) | 61.0(44.9-77.2) | 0.502 |
| **Dialysis** | 2,507(4.1%) | 911(4.2%) | 922(3.9%) | 674(4.2%) | 0.758 |
| **Hypertension** | 55,482(89.8%) | 19,669(90.1%) | 21,422(90.3%) | 14,391(88.8%) | <.001 |
| **Diabetes mellitus** | 23,375(37.9%) | 8,287(38.0%) | 8,896(37.5%) | 6,192(38.2%) | 0.787 |
| **Severe chronic lung disease** | 7,579(12.3%) | 2,835(13.0%) | 2,944(12.5%) | 1,800(11.2%) | <.001 |
| **Home oxygen** | 6,496(10.5%) | 2,334(10.7%) | 2,488(10.5%) | 1,674(10.3%) | 0.241 |
| **Hostile chest** | 4,437(7.2%) | 1,686(7.7%) | 1,459(6.2%) | 1,292(8.0%) | 0.892 |
| **Current/recent smoker** | 2,643(4.3%) | 905(4.1%) | 1,017(4.3%) | 721(4.4%) | 0.149 |
| **Cerebrovascular disease (stroke, TIA or carotid stenosis)** | 19,606(37.7%) | 7,187(39.6%) | 7,405(36.4%) | 5,014(37.2%) | <.001 |
| **Peripheral arterial disease** | 15,552(25.2%) | 5,722(26.2%) | 6,016(25.4%) | 3,814(23.5%) | <.001 |
| **Endocarditis** | 335(0.5%) | 124(0.6%) | 127(0.5%) | 84(0.5%) | 0.502 |
| **Permanent pacemaker** | 9,492(15.4%) | 3,392(15.5%) | 3,554(15.0%) | 2,546(15.7%) | 0.858 |
| **Previous ICD** | 2,519(4.1%) | 868(4.0%) | 993(4.2%) | 658(4.1%) | 0.584 |
| **Prior PCI** | 21,360(34.6%) | 7,491(34.3%) | 8,205(34.6%) | 5,664(35.0%) | 0.205 |
| **Prior CABG** | 15,212(24.6%) | 5,275(24.2%) | 5,785(24.4%) | 4,152(25.6%) | 0.002 |
| **Prior other cardiac surgery** | 3,014(4.9%) | 838(3.8%) | 1,340(5.7%) | 836(5.2%) | <.001 |
| **Prior aortic valve procedure** | 5,504(8.9%) | 1,722(7.9%) | 2,428(10.2%) | 1,354(8.4%) | 0.006 |
| **Prior non-aortic valve procedure** | 1,328(2.2%) | 510(2.3%) | 518(2.2%) | 300(1.9%) | 0.002 |
| **Prior MI** | 14,511(23.5%) | 5,087(23.3%) | 5,510(23.2%) | 3,914(24.2%) | 0.081 |
| **Heart failure within 2 weeks** | 48,124(78.0%) | 17,802(81.6%) | 18,758(79.1%) | 11,564(71.4%) | <.001 |
| **NYHA class IV within 2 weeks** | 10,393(16.9%) | 4,095(18.8%) | 4,021(17.1%) | 2,277(14.2%) | <.001 |
| **Porcelain aorta** | 2,565(4.2%) | 896(4.1%) | 900(3.8%) | 769(4.8%) | 0.009 |
| **Atrial fibrillation/flutter** | 24,831(40.2%) | 8,898(40.8%) | 9,441(39.8%) | 6,492(40.1%) | 0.113 |
| **Conduction defect** | 22,202(36.1%) | 8,237(37.9%) | 8,518(36.0%) | 5,447(33.8%) | <.001 |
| **Left main stenosis ≥50%** | 5,796(9.5%) | 1,986(9.2%) | 2,177(9.3%) | 1,633(10.2%) | 0.002 |
| **Proximal LAD stenosis ≥70%** | 11,545(18.9%) | 3,988(18.4%) | 4,305(18.3%) | 3,252(20.3%) | <.001 |
| **LVEF (%)** | 58.0(48.0-64.0) | 58.0(48.0-65.0) | 58.0(47.0-64.0) | 58.0(48.0-63.0) | <.001 |
| **Degenerative aortic valve disease** | 59,265(96.1%) | 20,761(95.1%) | 23,120(97.5%) | 15,384(95.1%) | 0.059 |
| **Tricuspid aortic valve** | 55,717(90.6%) | 20,190(92.8%) | 20,575(87.2%) | 14,952(92.8%) | 0.002 |
| **Moderate/severe aortic insufficiency** | 11,082(18.0%) | 3,745(17.2%) | 4,389(18.6%) | 2,948(18.3%) | 0.002 |
| **Moderate/severe mitral insufficiency** | 17,037(27.7%) | 6,037(27.7%) | 6,559(27.8%) | 4,441(27.5%) | 0.653 |
| **Moderate/Severe tricuspid insufficiency** | 14,489(23.6%) | 5,142(23.7%) | 5,638(23.9%) | 3,709(23.1%) | 0.201 |
| **Elective procedure** | 56,691(91.8%) | 19,942(91.4%) | 21,858(92.1%) | 14,891(91.8%) | 0.047 |

Abbreviations: CABG – coronary artery bypass grafting; GFR – glomerular filtration rate; ICD – implantable cardioverter-defibrillator; LAD – left anterior descending; LVEF – left ventricular ejection fraction; MDRD – modification of diet in renal disease; MI – myocardial infarction; NYHA – New York Heart Association; PCI – percutaneous coronary intervention; PROM – predicted risk of mortality, STS – Society of Thoracic Surgeons; TIA – transient ischemic attack

**Supplementary Table 2:** Unadjusted post-TAVR mortality rates stratified by hospital mortality tertile.

| **Tertile** | **N (hospitals)** | **N (patients)** | **Post-TAVR deaths (%)** | **O/E Mortality Ratio Range** |
| --- | --- | --- | --- | --- |
| Lowest | 143 | 21842 | 393 (1.8%) | 0.000-0.335 |
| Middle | 143 | 23743 | 795 (3.3%) | 0.336-0.578 |
| Highest | 143 | 16219 | 913 (5.6%) | 0.579-2.432 |

**Supplementary Table 3**: Observed complication rates stratified by hospital mortality tertile.

|  | **Overall  N=61,804** | **Lowest Tertile  N=21,842** | **Middle Tertile  N=23,743** | **Highest Tertile  N=16,219** | ***p*-value** |
| --- | --- | --- | --- | --- | --- |
| **New requirement for pacemaker or ICD** | 6,749(10.9%) | 2,235(10.2%) | 2,773(11.7%) | 1,741(10.7%) | 0.030 |
| **Major vascular complication** | 4,337(7.0%) | 1,438(6.6%) | 1,616(6.8%) | 1,283(7.9%) | <.001 |
| **Conversion to open surgery** | 334(0.5%) | 97(0.4%) | 135(0.6%) | 102(0.6%) | 0.012 |
| **Stroke** | 1,084(1.8%) | 351(1.6%) | 410(1.7%) | 323(2.0%) | 0.006 |
| **Cardiac arrest** | 1,540(2.5%) | 433(2.0%) | 612(2.6%) | 495(3.1%) | <.001 |
| **Aortic-related complication** | 803(1.3%) | 229(1.0%) | 309(1.3%) | 265(1.6%) | <.001 |
| **Device-related complication** | 651(1.1%) | 201(0.9%) | 277(1.2%) | 173(1.1%) | 0.097 |
| **New requirement for dialysis** | 509(0.8%) | 143(0.7%) | 199(0.8%) | 167(1.0%) | <.001 |
| **Unplanned other cardiac surgery or intervention** | 736(1.2%) | 232(1.1%) | 275(1.2%) | 229(1.4%) | 0.003 |
| **VARC major or life-threatening bleeding** | 4,142(6.7%) | 1,397(6.4%) | 1,514(6.4%) | 1,231(7.6%) | <.001 |
| **Composite of all complications** | 15,033(24.3%) | 4,952(22.7%) | 5,843(24.6%) | 4,238(26.1%) | <.001 |

Abbreviations: ICD – implantable cardioverter defibrillator; VARC – Valve Academic Research Consortium

**Supplemental Table 4.**  Unadjusted complication-specific FTR rates by hospital mortality tertile.

|  | **Overall  N=61,804** | **Lowest Tertile  N=21,842** | **Middle Tertile  N=23,743** | **Highest Tertile  N=16,219** | ***p*-value** |
| --- | --- | --- | --- | --- | --- |
| **FTR from new requirement for pacemaker or ICD** | 251 (3.7%) | 50 (2.2%) | 101 (3.6%) | 100 (5.7%) | <0.001 |
| **FTR from major vascular complication** | 417 (9.6%) | 70 (4.9%) | 170 (10.5%) | 177 (13.8%) | <0.001 |
| **FTR from conversion to open surgery** | 145 (43.4%) | 23 (23.7%) | 60 (44.4%) | 62 (60.8%) | <0.001 |
| **FTR from stroke** | 205 (18.9%) | 39 (11.1%) | 73 (17.8%) | 93 (28.8%) | <0.001 |
| **FTR from cardiac arrest** | 641 (41.6%) | 129 (29.8%) | 241 (39.4%) | 271 (54.7%) | <0.001 |
| **FTR from aortic-related complication** | 228 (28.4%) | 42 (18.3%) | 92 (29.8%) | 94 (35.5%) | <0.001 |
| **FTR from device-related complication** | 121 (18.6%) | 25 (12.4%) | 49 (17.7%) | 47 (27.2%) | <0.001 |
| **FTR from new requirement for dialysis** | 240 (47.2%) | 48 (33.6%) | 82 (41.2%) | 110 (65.9%) | <0.001 |
| **FTR from unplanned other cardiac surgery or intervention** | 202 (27.4%) | 40 (17.2%) | 83 (30.2%) | 79 (34.5%) | <0.001 |
| **FTR from VARC major or life-threatening bleeding** | 523 (12.6%) | 89 (6.4%) | 196 (12.9%) | 238 (19.3%) | <0.001 |
| **FTR from composite of all complications** | 1,447 (9.6%) | 263 (5.3%) | 557 (9.5%) | 627 (14.8%) | <0.001 |

Note: the denominator for calculating a complication-specific FTR rate is the number of patients with the complication and so varies across table cells. Abbreviations: FTR – failure to rescue; ICD – implantable cardioverter defibrillator; VARC – Valve Academic Research Consortium

**Supplementary Table 5:** Adjusted associations between hospital mortality tertiles and complications.

|  | | **Adjusted** | |
| --- | --- | --- | --- |
| **Outcome** |  | **Odds Ratio (95% CI)** | ***p*-value** |
| New requirement for pacemaker or ICD | Middle vs. Lowest Tertile | 1.19 (1.04-1.36) | 0.009 |
|  | Highest vs. Lowest Tertile | 1.17 (1.02-1.34) | 0.026 |
|  | Hospital TAVR Volume (per 20 cases) | 1.01 (1.00-1.01) | 0.005 |
|  | Teaching Hospital | 1.11 (0.98-1.26) | 0.087 |
|  | 501-1000 vs. 0-500 Beds | 1.03 (0.91-1.16) | 0.677 |
|  | >1000 vs. 0-500 Beds | 1.08 (0.81-1.43) | 0.604 |
| Significant vascular complication | Middle vs. Lowest Tertile | 1.03 (0.88-1.21) | 0.717 |
|  | Highest vs. Lowest Tertile | 1.31 (1.11-1.54) | 0.001 |
|  | Hospital TAVR Volume (per 20 cases) | 1.00 (0.99-1.01) | 0.893 |
|  | Teaching Hospital | 1.05 (0.90-1.21) | 0.562 |
|  | 501-1000 vs. 0-500 Beds | 0.91 (0.79-1.06) | 0.228 |
|  | >1000 vs. 0-500 Beds | 0.93 (0.65-1.32) | 0.669 |
| Conversion to open heart surgery | Middle vs. Lowest Tertile | 1.29 (0.96-1.73) | 0.086 |
|  | Highest vs. Lowest Tertile | 1.35 (0.99-1.84) | 0.059 |
|  | Hospital TAVR Volume (per 20 cases) | 0.98 (0.97-0.99) | 0.001 |
|  | Teaching Hospital | 1.09 (0.82-1.45) | 0.553 |
|  | 501-1000 vs. 0-500 Beds | 1.22 (0.93-1.60) | 0.161 |
|  | >1000 vs. 0-500 Beds | 1.38 (0.78-2.42) | 0.267 |
| Stroke* | Middle vs. Lowest Tertile | 1.14 (0.94-1.37) | 0.179 |
|  | Highest vs. Lowest Tertile | 1.35 (1.11-1.65) | 0.002 |
|  | Hospital TAVR Volume (per 20 cases) | 1.00 (0.99-1.01) | 0.543 |
|  | Teaching Hospital | 0.98 (0.82-1.17) | 0.835 |
|  | 501-1000 vs. 0-500 Beds | 0.99 (0.83-1.18) | 0.902 |
|  | >1000 vs. 0-500 Beds | 1.07 (0.74-1.55) | 0.711 |
| Cardiac arrest | Middle vs. Lowest Tertile | 1.34 (1.13-1.57) | <.001 |
|  | Highest vs. Lowest Tertile | 1.63 (1.37-1.93) | <.001 |
|  | Hospital TAVR Volume (per 20 cases) | 0.99 (0.99-1.00) | 0.067 |
|  | Teaching Hospital | 1.04 (0.89-1.22) | 0.598 |
|  | 501-1000 vs. 0-500 Beds | 1.00 (0.86-1.17) | 0.950 |
|  | >1000 vs. 0-500 Beds | 1.01 (0.72-1.40) | 0.968 |
| Aortic related complication | Middle vs. Lowest Tertile | 1.26 (1.03-1.54) | 0.027 |
|  | Highest vs. Lowest Tertile | 1.55 (1.26-1.92) | <.001 |
|  | Hospital TAVR Volume (per 20 cases) | 0.99 (0.98-1.00) | 0.007 |
|  | Teaching Hospital | 0.96 (0.79-1.16) | 0.661 |
|  | 501-1000 vs. 0-500 Beds | 1.16 (0.96-1.40) | 0.122 |
|  | >1000 vs. 0-500 Beds | 1.09 (0.72-1.65) | 0.680 |
| Device related complication | Middle vs. Lowest Tertile | 1.28 (0.98-1.67) | 0.069 |
|  | Highest vs. Lowest Tertile | 1.29 (0.97-1.72) | 0.083 |
|  | Hospital TAVR Volume (per 20 cases) | 1.01 (1.00-1.02) | 0.173 |
|  | Teaching Hospital | 0.96 (0.74-1.23) | 0.729 |
|  | 501-1000 vs. 0-500 Beds | 0.79 (0.62-1.02) | 0.069 |
|  | >1000 vs. 0-500 Beds | 1.03 (0.61-1.76) | 0.904 |
| New requirement for dialysis | Middle vs. Lowest Tertile | 1.29 (1.00-1.66) | 0.054 |
|  | Highest vs. Lowest Tertile | 1.68 (1.28-2.20) | <.001 |
|  | Hospital TAVR Volume (per 20 cases) | 1.00 (0.99-1.01) | 0.907 |
|  | Teaching Hospital | 1.14 (0.89-1.46) | 0.291 |
|  | 501-1000 vs. 0-500 Beds | 1.02 (0.80-1.29) | 0.898 |
|  | >1000 vs. 0-500 Beds | 1.03 (0.64-1.66) | 0.913 |
| Unplanned other cardiac surgery or intervention | Middle vs. Lowest Tertile | 1.02 (0.79-1.32) | 0.855 |
|  | Highest vs. Lowest Tertile | 1.33 (1.02-1.73) | 0.037 |
|  | Hospital TAVR Volume (per 20 cases) | 1.00 (0.99-1.01) | 0.918 |
|  | Teaching Hospital | 0.94 (0.73-1.19) | 0.593 |
|  | 501-1000 vs. 0-500 Beds | 1.26 (0.99-1.59) | 0.061 |
|  | >1000 vs. 0-500 Beds | 1.27 (0.75-2.14) | 0.371 |
| VARC major or life threatening bleeding | Middle vs. Lowest Tertile | 1.03 (0.87-1.22) | 0.744 |
|  | Highest vs. Lowest Tertile | 1.28 (1.07-1.52) | 0.006 |
|  | Hospital TAVR Volume (per 20 cases) | 1.00 (0.99-1.00) | 0.280 |
|  | Teaching Hospital | 1.08 (0.92-1.26) | 0.367 |
|  | 501-1000 vs. 0-500 Beds | 0.99 (0.85-1.16) | 0.939 |
|  | >1000 vs. 0-500 Beds | 0.94 (0.64-1.38) | 0.751 |
| Composite of all complications | Middle vs. Lowest Tertile | 1.14 (1.04-1.26) | 0.007 |
|  | Highest vs. Lowest Tertile | 1.31 (1.19-1.45) | <.001 |
|  | Hospital TAVR Volume (per 20 cases) | 1.00 (1.00-1.01) | 0.393 |
|  | Teaching Hospital | 1.07 (0.98-1.17) | 0.126 |
|  | 501-1000 vs. 0-500 Beds | 1.00 (0.91-1.09) | 0.938 |
|  | >1000 vs. 0-500 Beds | 1.00 (0.81-1.24) | 0.984 |

*Endocarditis was removed from the multivariable model for stroke to avoid convergence issues.

Abbreviations: ICD – implantable cardioverter defibrillator; TAVR – transcatheter aortic valve replacement; VARC – Valve Academic Research Consortium

**Supplementary Table 6:** Unadjusted and adjusted associations between hospital mortality tertiles and FTR from complications.

|  | | **Unadjusted** | | **Adjusted** | |
| --- | --- | --- | --- | --- | --- |
| **Outcome** |  | **Odds Ratio (95% CI)** | ***p*-value** | **Odds Ratio (95% CI)** | ***p*-value** |
| FTR from New Requirement for pacemaker or ICD^a^ | Middle vs. Lowest Tertile | 1.69 (1.18-2.43) | 0.004 | 1.71 (1.18-2.47) | 0.004 |
|  | Highest vs. Lowest Tertile | 2.73 (1.90-3.91) | <.001 | 2.74 (1.87-4.00) | <.001 |
|  | Hospital TAVR Volume (per 20 cases) |  |  | 0.99 (0.98-1.01) | 0.401 |
|  | Teaching Hospital |  |  | 0.89 (0.65-1.22) | 0.457 |
|  | 501-1000 vs. 0-500 Beds |  |  | 1.14 (0.84-1.55) | 0.404 |
|  | >1000 vs. 0-500 Beds |  |  | 0.33 (0.12-0.85) | 0.022 |
| FTR from Significant Vascular Complication | Middle vs. Lowest Tertile | 2.30 (1.72-3.06) | <.001 | 2.32 (1.72-3.13) | <.001 |
|  | Highest vs. Lowest Tertile | 3.13 (2.35-4.17) | <.001 | 3.35 (2.46-4.56) | <.001 |
|  | Hospital TAVR Volume (per 20 cases) |  |  | 1.00 (0.99-1.01) | 0.916 |
|  | Teaching Hospital |  |  | 1.00 (0.78-1.29) | 0.995 |
|  | 501-1000 vs. 0-500 Beds |  |  | 0.84 (0.66-1.07) | 0.167 |
|  | >1000 vs. 0-500 Beds |  |  | 0.91 (0.55-1.49) | 0.702 |
| FTR from Conversion to Open Heart Surgery^b^ | Middle vs. Lowest Tertile | 2.57 (1.44-4.61) | 0.002 | 2.99 (1.48-6.07) | 0.003 |
|  | Highest vs. Lowest Tertile | 4.99 (2.68-9.27) | <.001 | 9.04 (4.12-19.83) | <.001 |
|  | Hospital TAVR Volume (per 20 cases) |  |  | 1.01 (0.98-1.04) | 0.569 |
|  | Teaching Hospital |  |  | 0.82 (0.42-1.62) | 0.570 |
|  | 501-1000 vs. 0-500 Beds |  |  | 1.15 (0.59-2.25) | 0.683 |
|  | >1000 vs. 0-500 Beds |  |  | 1.01 (0.28-3.59) | 0.991 |
| FTR from Stroke^c^ | Middle vs. Lowest Tertile | 1.74 (1.13-2.67) | 0.012 | 1.67 (1.05-2.67) | 0.032 |
|  | Highest vs. Lowest Tertile | 3.28 (2.15-5.00) | <.001 | 3.15 (1.97-5.04) | <.001 |
|  | Hospital TAVR Volume (per 20 cases) |  |  | 0.99 (0.97-1.01) | 0.286 |
|  | Teaching Hospital |  |  | 1.24 (0.83-1.87) | 0.298 |
|  | 501-1000 vs. 0-500 Beds |  |  | 0.76 (0.51-1.13) | 0.169 |
|  | >1000 vs. 0-500 Beds |  |  | 1.13 (0.54-2.39) | 0.741 |
| FTR from Cardiac Arrest | Middle vs. Lowest Tertile | 1.54 (1.17-2.03) | 0.002 | 1.67 (1.24-2.24) | <.001 |
|  | Highest vs. Lowest Tertile | 2.89 (2.18-3.84) | <.001 | 3.54 (2.57-4.87) | <.001 |
|  | Hospital TAVR Volume (per 20 cases) |  |  | 1.01 (1.00-1.02) | 0.055 |
|  | Teaching Hospital |  |  | 0.81 (0.62-1.07) | 0.146 |
|  | 501-1000 vs. 0-500 Beds |  |  | 1.15 (0.88-1.52) | 0.301 |
|  | >1000 vs. 0-500 Beds |  |  | 1.07 (0.61-1.87) | 0.819 |
| FTR from Aortic Related Complication^d^ | Middle vs. Lowest Tertile | 1.89 (1.25-2.86) | 0.003 | 2.04 (1.31-3.18) | 0.002 |
|  | Highest vs. Lowest Tertile | 2.45 (1.61-3.72) | <.001 | 3.07 (1.92-4.89) | <.001 |
|  | Hospital TAVR Volume (per 20 cases) |  |  | 1.01 (0.99-1.02) | 0.423 |
|  | Teaching Hospital |  |  | 1.20 (0.79-1.81) | 0.393 |
|  | 501-1000 vs. 0-500 Beds |  |  | 1.09 (0.74-1.63) | 0.655 |
|  | >1000 vs. 0-500 Beds |  |  | 1.19 (0.54-2.61) | 0.660 |
|  |  |  |  |  |  |
| FTR from Device Related Complication^c^ | Middle vs. Lowest Tertile | 1.51 (0.90-2.55) | 0.120 | 1.45 (0.80-2.62) | 0.220 |
|  | Highest vs. Lowest Tertile | 2.63 (1.53-4.50) | <.001 | 2.90 (1.53-5.50) | 0.001 |
|  | Hospital TAVR Volume (per 20 cases) |  |  | 0.98 (0.96-1.00) | 0.107 |
|  | Teaching Hospital |  |  | 1.37 (0.77-2.41) | 0.282 |
|  | 501-1000 vs. 0-500 Beds |  |  | 0.74 (0.43-1.27) | 0.279 |
|  | >1000 vs. 0-500 Beds |  |  | 0.46 (0.15-1.43) | 0.179 |
| FTR from New Requirement for Dialysis^c^ | Middle vs. Lowest Tertile | 1.41 (0.85-2.34) | 0.182 | 1.58 (0.84-2.97) | 0.155 |
|  | Highest vs. Lowest Tertile | 4.15 (2.44-7.05) | <.001 | 6.16 (3.08-12.31) | <.001 |
|  | Hospital TAVR Volume (per 20 cases) |  |  | 1.03 (1.01-1.06) | 0.009 |
|  | Teaching Hospital |  |  | 0.76 (0.42-1.37) | 0.354 |
|  | 501-1000 vs. 0-500 Beds |  |  | 1.30 (0.73-2.34) | 0.374 |
|  | >1000 vs. 0-500 Beds |  |  | 0.44 (0.14-1.40) | 0.163 |
| FTR from Unplanned Other Cardiac Surgery or Intervention | Middle vs. Lowest Tertile | 2.13 (1.36-3.34) | 0.001 | 2.34 (1.43-3.84) | <.001 |
|  | Highest vs. Lowest Tertile | 2.56 (1.62-4.04) | <.001 | 3.06 (1.83-5.14) | <.001 |
|  | Hospital TAVR Volume (per 20 cases) |  |  | 1.00 (0.99-1.02) | 0.704 |
|  | Teaching Hospital |  |  | 0.92 (0.59-1.43) | 0.699 |
|  | 501-1000 vs. 0-500 Beds |  |  | 0.91 (0.59-1.38) | 0.643 |
|  | >1000 vs. 0-500 Beds |  |  | 0.61 (0.23-1.62) | 0.322 |
| FTR from VARC Major or Life Threatening Bleeding | Middle vs. Lowest Tertile | 2.23 (1.69-2.93) | <.001 | 2.24 (1.71-2.95) | <.001 |
|  | Highest vs. Lowest Tertile | 3.63 (2.77-4.75) | <.001 | 3.83 (2.90-5.06) | <.001 |
|  | Hospital TAVR Volume (per 20 cases) |  |  | 1.00 (0.99-1.01) | 0.694 |
|  | Teaching Hospital |  |  | 0.73 (0.59-0.92) | 0.008 |
|  | 501-1000 vs. 0-500 Beds |  |  | 1.01 (0.81-1.27) | 0.932 |
|  | >1000 vs. 0-500 Beds |  |  | 0.97 (0.59-1.60) | 0.915 |
| FTR from Composite of All Complications | Middle vs. Lowest Tertile | 1.88 (1.61-2.19) | <.001 | 1.90 (1.63-2.22) | <.001 |
|  | Highest vs. Lowest Tertile | 3.10 (2.66-3.60) | <.001 | 3.16 (2.69-3.70) | <.001 |
|  | Hospital TAVR Volume (per 20 cases) |  |  | 1.00 (0.99-1.00) | 0.100 |
|  | Teaching Hospital |  |  | 0.90 (0.79-1.03) | 0.133 |
|  | 501-1000 vs. 0-500 Beds |  |  | 0.99 (0.87-1.13) | 0.905 |
|  | >1000 vs. 0-500 Beds |  |  | 0.89 (0.68-1.18) | 0.430 |

^a^Permanent pacemaker was removed from the multivariable model for FTR from new requirement for pacemaker or ICD to avoid convergence issues.

^b^Endocarditis, prior non-aortic valve procedure, and one of the TAVR acuity categories (emergency or salvage or cardiac arrest) were removed from the multivariable model for FTR from conversion to open heart surgery to avoid convergence issues.

^c^Endocarditis was removed from the multivariable models for FTR from stroke, device related complication, and new requirement for dialysis to avoid convergence issues.

^d^Endocarditis and prior non-aortic valve procedure were removed from the multivariable model for FTR from aortic related complication to avoid convergence issues.

Abbreviations: FTR – failure to rescue; ICD – implantable cardioverter defibrillator; TAVR – transcatheter aortic valve replacement
